# Esthetic Rehabilitation of the Smile with No-Prep Porcelain Laminates and Partial Veneers

**DOI:** 10.1155/2015/452765

**Published:** 2015-10-18

**Authors:** Arcelino Farias-Neto, Edna Maria da Cunha Ferreira Gomes, Alfonso Sánchez-Ayala, Alejandro Sánchez-Ayala, Larissa Soares Reis Vilanova

**Affiliations:** ^1^Department of Dentistry, Health School, Potiguar University, Laureate International Universities, Avenida Senador Salgado Filho, No. 1610, 59056-000 Natal, RN, Brazil; ^2^Department of Dentistry, State University of Ponta Grossa, Avenida General Carlos Cavalcanti, No. 4748, 84030-900 Ponta Grossa, PR, Brazil; ^3^Department of Dentistry, University of San Martín de Porres, Jr. Las Calandrias 151-291, Lima 43, Peru; ^4^Department of Oral Health, Federal University of Goiás, Primeira Avenida, s/n, 74605-020 Goiânia, GO, Brazil

## Abstract

Rehabilitation of patients with anterior conoid teeth may present a challenge for the clinician, especially when trying to mimic the nature with composite resins. This clinical report exemplifies how a patient with conoid upper lateral incisors was rehabilitated with minimally invasive adhesive restorations. Following diagnostic wax-up and cosmetic mock-up, no-prep veneers and ceramic fragments (partial veneers) were constructed with feldspathic porcelain. This restorative material presents excellent reproduction of the optical properties of the dental structure, especially at minimal thicknesses. In this paper, the details about the treatment are described. A very pleasing outcome was achieved, confirming that minimally invasive adhesive restorations are an excellent option for situations in which the dental elements are healthy, and can be modified exclusively by adding material and the patient does not want to suffer any wear on the teeth.

## 1. Introduction

Nowadays, cosmetic needs are of fundamental importance to much of society. Among the available esthetic restorative materials, professionals have options ranging from composite resins to ceramics. For a long time, the material of choice for cosmetic and conservative procedures was composite resin. However, the low durability of this material leads to esthetic damage due to color instability. In addition, its organic matrix degrades and it absorbs water; therefore, the material needs constant maintenance and polishing to prolong the duration of its useful life. Porcelain greatly mimics the natural structure of dental elements and is an excellent option to avoid the various deficiencies of composite resin [1]. When properly made in accordance with a precise clinical protocol, porcelain restorations have a long clinical life. The material has several important characteristics, including physicochemical stability, excellent biological compatibility, sufficient resistance to compression and abrasion, excellent reproduction of the optical properties of the dental structure, adherence to the cement agent and dental substrates, and color stability [2].

The idea behind minimally invasive cosmetic dentistry is that the clinician should choose the most conservative method possible, thereby avoiding unnecessary wear and tear on the dental structure, while restoring function and appearance to the patient. The development of minimally invasive dentistry was only possible thanks to technological advances in ceramic systems and the development of the adhesive cementation technique. Initially, dentists cemented 0.5 mm thick laminate veneers to an unprepared dental surface. The material used was feldspathic ceramic, which has good clinical and laboratory sensitivity, especially at minimal thicknesses. However, gum inflammation was observed over time after cementation because of the overcontour created by these restorations. Therefore, dentists opted to limit tooth preparations to the space required for these restorations, so as to develop the original emergence profile of the teeth [3]. The perfecting of current ceramic systems, especially pressed ceramics reinforced with lithium disilicate, has brought us back to the idea of no-prep veneers. Although these veneers achieve thicknesses similar to those of feldspathic ceramics, lithium disilicate ceramics allow for restorations of up to 0.2 mm in thickness with greater clinical and laboratory ease. Because of their better mechanical properties, these restorations can be made, finished, tested, and cemented more safely [4]. This clinical report presents the case of a patient with conoid upper lateral incisors who was rehabilitated with no-prep veneers and ceramic fragments.

## 2. Case Report 

**Figure 1:**
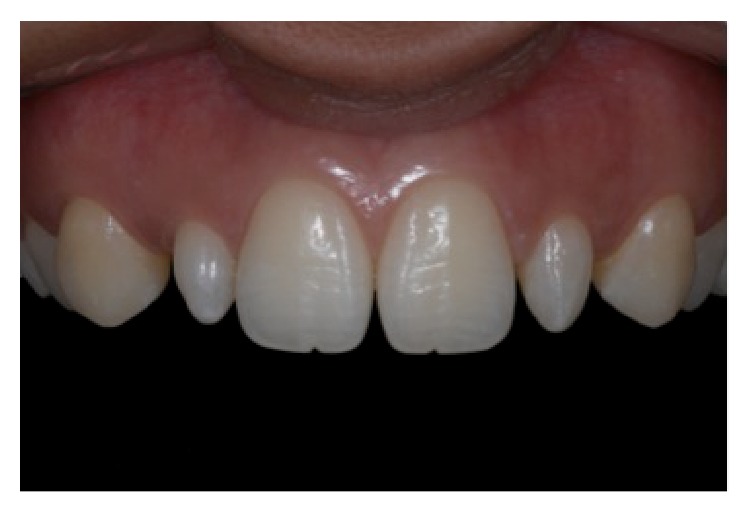
Intraoral front view, reprinted with permission.

**Figure 2 fig2:**
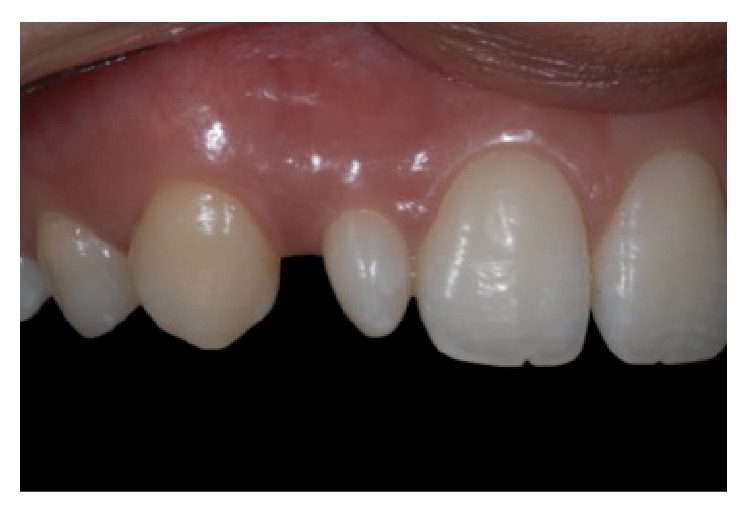
Intraoral right lateral view, reprinted with permission.

**Figure 3:**
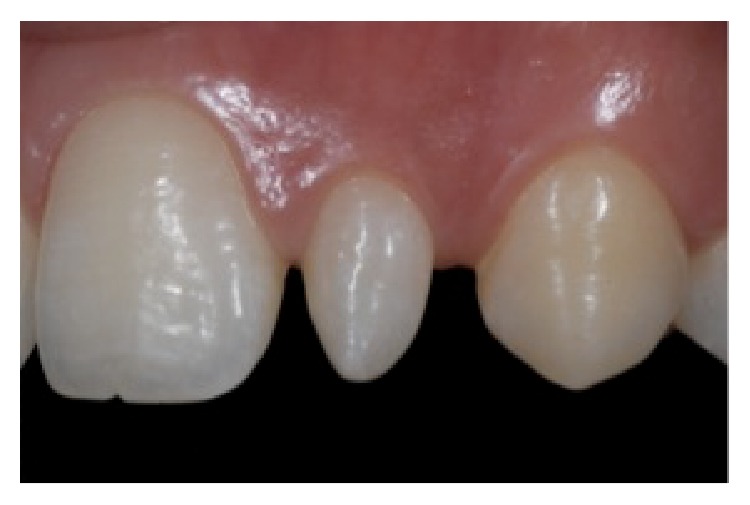
Intraoral left lateral view, reprinted with permission.

**Figure 4:**
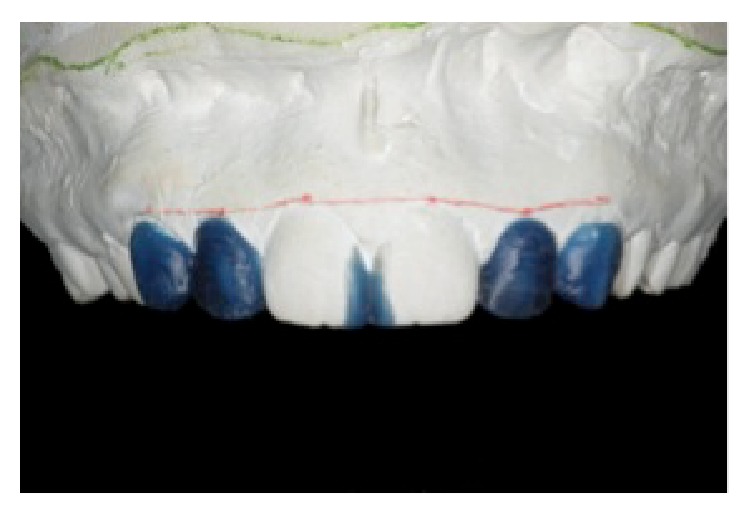
Diagnostic wax-up, reprinted with permission.

A 19-year-old female patient presented with conoid upper lateral incisors. During anamnesis, she reported dissatisfaction with her appearance. At the first visit, intraoral photographs were obtained to analyze the cosmetic aspects of the case (Figures 1 [Fig fig2]–3). During the second session, alginate impressions (Hydrogum 5, Zhermack, Badia Polesine, RO, Italy) of the upper and lower dental arches were obtained. The dental casts were mounted in a semiadjustable articulator and sent, together with the photographs, to the dental laboratory for diagnostic wax mold (Figure 4). This information helped the dental technician achieve a better and more detailed cosmetic analysis without requiring the presence of the patient. To obtain an acceptable reconstruction from the cosmetic and functional perspectives, a diagnostic wax-up of the upper model was made in blue wax. Due to the excessive space between the upper conoid lateral incisors and the canines, both teeth were covered in wax, to avoid the need to reconstruct very large lateral incisors. In addition, the mesial-incisal angle of the upper central incisors was covered in wax to reduce the incisal embrasure and obtain a more harmonious smile.

**Figure 5:**
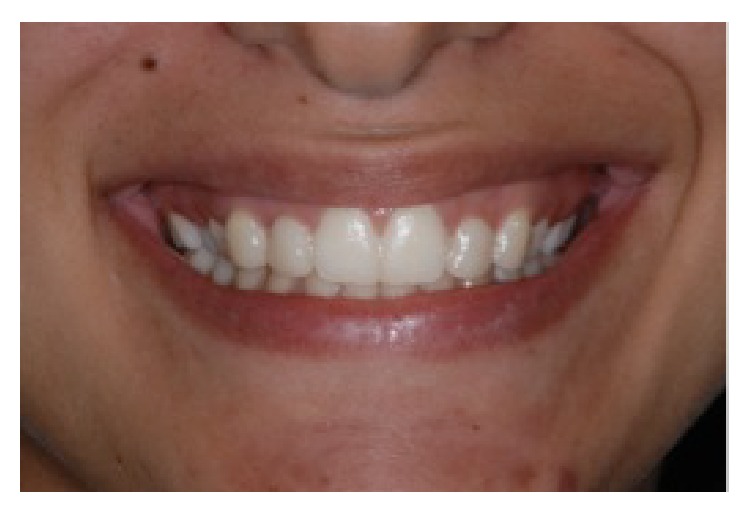
View of the smile with the cosmetic mock-up, reprinted with permission.

**Figure 6:**
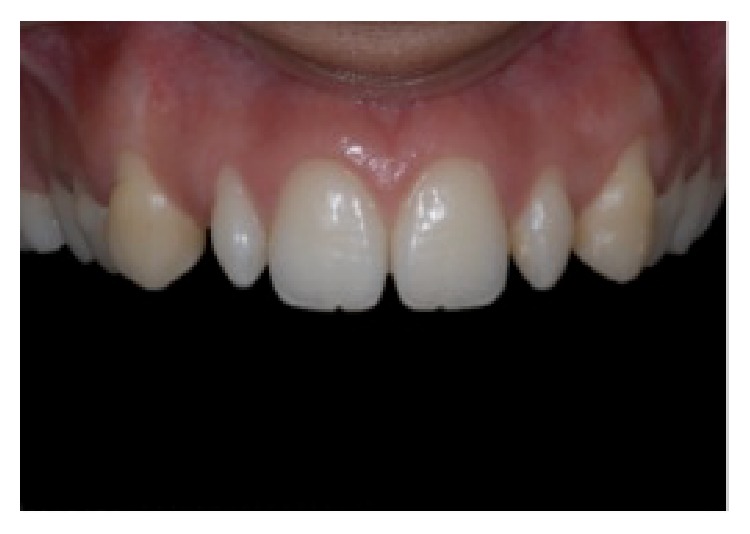
Appearance 4 months after clinical crown lengthening to correct the gummy smile, reprinted with permission.

A cosmetic mock-up was accomplished to give the patient a three-dimensional view of her new smile before starting treatment. First, the diagnostic wax-up was molded with condensation silicon to generate a matrix. This silicon matrix was filled with a bis-acryl resin (Structur 2 SC, Voco, Porto Alegre, RS, Brazil), which was positioned over the dental elements and maintained in position for approximately 2 minutes. After the silicon matrix was removed, the mock-up (artificial resin shell) remained mechanically attached to the teeth (Figure 5). The cosmetic mock-up enabled a three-dimensional analysis of the new dental proportions together with the soft tissues (lips and gum). The analysis of the new smile with the mock-up made the patient's gummy smile more obvious. Thus, before beginning restorative treatment, clinical crown lengthening was performed (Figure 6). Bone resection was performed to reestablish appropriate biological width, gingival zenith, clinical crown length/width ratio, and gum exposure during smile. A period of 4 months was necessary to achieve periodontal tissue healing and gum line stabilization before final impression.

**Figure 7:**
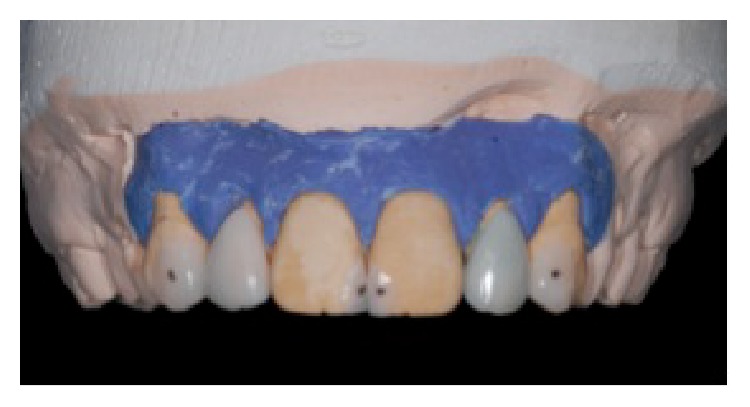
No-prep veneers for upper lateral incisors and ceramic fragments for canines and central incisors, reprinted with permission.

**Figure 8:**
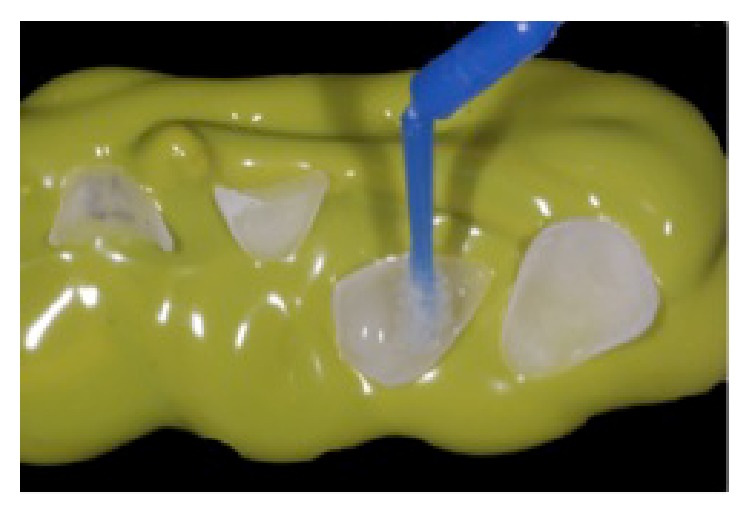
Application of silane agent for 1 minute. Silane was rubbed for 10 seconds, followed by evaporation of the solvent for 1 minute, reprinted with permission.

**Figure 9:**
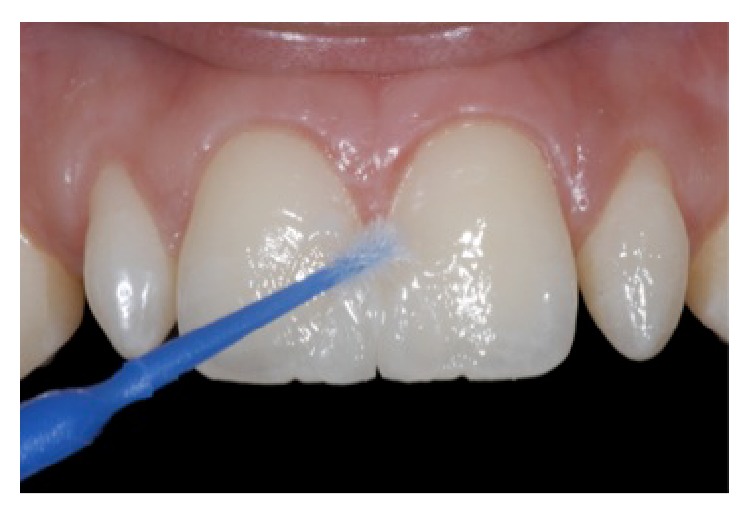
Removal of excess cement with a disposable brush and dental floss before light activation. Care should be taken so that the fragments do not change position, reprinted with permission.

**Figure 10:**
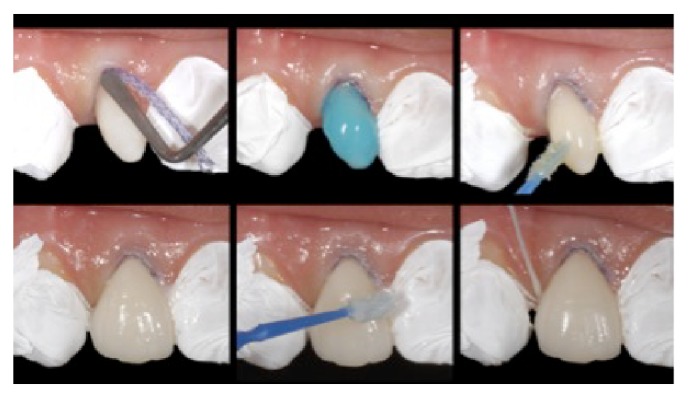
Cementation of the no-prep veneer on the upper right lateral incisor. Adjacent teeth were protected with thread-seal tape. Insertion of gingival retraction cord, conditioning of the enamel with 37% phosphoric acid for 30 seconds, and application of the adhesive system without light activation. Excess cement was removed with a brush and dental floss before light activation, reprinted with permission.

A polyvinyl siloxane impression (Express XT, 3M, Sumaré, SP, Brazil) was made to build the ceramic restorations. Gingival retraction cord (Ultrapak, Ultradent, Indaiatuba, SP, Brazil) impregnated with aluminum chloride (Hemostop, Dentsply, Petrópolis, RJ, Brazil) was used on the lateral incisors. Despite the absence of a finish line preparation, gingival retraction cord was used to obtain a better emergence profile for the veneers. Using the information obtained from the diagnostic wax-up and the mock-up, the dental laboratory constructed ceramic no-prep veneers for the upper maxillary lateral incisors and ceramic fragments (no-prep partial veneers) for canines and central incisors (Figure 7). All restorations were constructed with feldspathic porcelain. Before cementation, the restorations were evaluated in terms of adaptation, and the color of the adhesive cement was selected by the use of a try-in cement (Allcem Veneer Try-in, FGM, Joinville, SC, Brazil). A transparent color was selected for the central incisors and color A2 for the lateral incisors and canines. Before cementation, the restorations were thoroughly washed to eliminate the try-in cement. The following precementation surface treatment was applied (Table 1): etching with 10% hydrofluoric acid (Condac Porcelana, FGM, Joinville, SC, Brazil) for 90 seconds, washing, drying, application of silane agent (Prosil, FGM) for 1 minute (Figure 8), and application of adhesive (Ambar, FGM, Joinville, SC, Brazil) (no light activation). Prophylaxis was performed on the dental structure with a Robinson brush, pumice paste, and water (Table 2). The tooth surface was etched with 37% phosphoric acid (Condac 37, FGM, Joinville, SC, Brazil) for 30 seconds, followed by washing with a water and air jet. Excess water was removed to keep the enamel surface dry. The adhesive was applied to the tooth without light activation. The restorations were cemented with a photopolymerizable adhesive cement (Allcem Veneer, FGM, Joinville, SC, Brazil) using the previously selected colors. Because no dental preparation was performed, the ceramic fragments of the central incisors were cemented at the same time for a more precise positioning. If you cement one at a time, a wrong position may affect contact point and midline. When you cement both central incisors at the same, you have a more accurate analysis of both tooth contours (Figure 9). The remaining pieces were cemented individually, while the adjacent teeth were protected with thread-seal tape (Figure 10).

**Figure 11:**
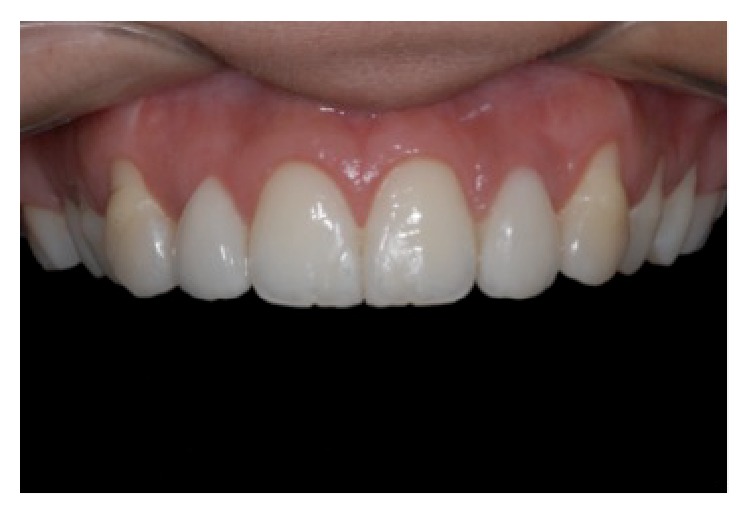
Front view of the result of the first phase of treatment. Observe the presence of exposed roots on the upper canines (consequence of the clinical crown lengthening procedure), lingualization of elements upper right premolars, and the rotation of the canine, reprinted with permission.

**Figure 12:**
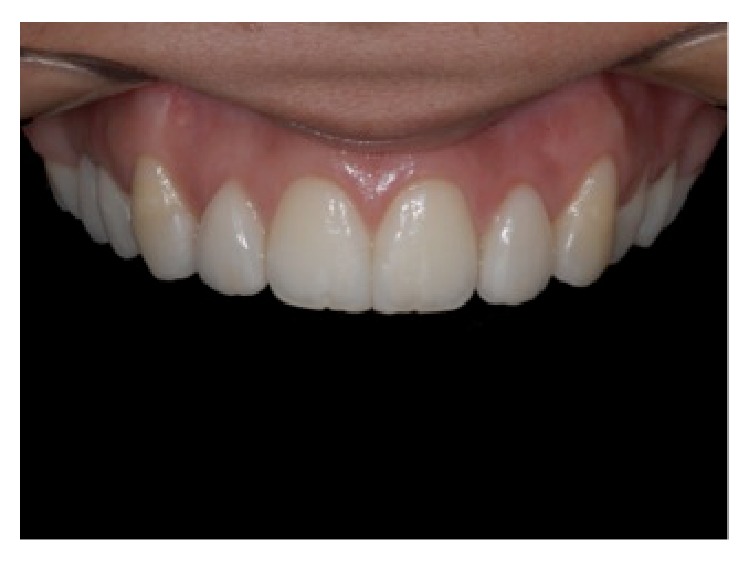
Close-up view of the upper incisors. Presence of ceramic fragments on the mesial surfaces of elements central incisors and no-prep veneers on lateral incisors, reprinted with permission.

Final occlusal adjustment was done away from the relative isolation of the operating field, when the postglazing polishing of the restoration with rubber polishing points was also performed.  Figure 11 shows the results of this first treatment phase. In the second phase of the treatment, ceramic fragments were constructed to cover the root exposure present in the upper canines (a consequence of the clinical crown lengthening procedure). Additionally, the buccal corridor on the right side (lingualization of the first and second upper premolars) was corrected with no-prep veneers (Figure 11), and enameloplasty on the distal surface of right upper canine was accomplished (Figure 11).  Figure 12 shows the final result.

## 3. Discussion 

The patient's main complaint at the start of treatment was her cosmetic discomfort with the presence of conoid upper lateral incisors. However, even the main rules of the golden ratio may not be able to turn into reality every patient's subjective desire. In cosmetic oral rehabilitation procedures, final restorations should not be made until the patient has had a chance to preview the treatment and state that it meets his or her expectations. In this case, before performing any irreversible procedure, a real three-dimensional visualization of the final shape of the proposed treatment was achieved by the cosmetic mock-up. While the diagnostic wax-up represented only the desired shape of the teeth (Figure 4), the mock-up went further because it visualized the patient's smile, integrating the gum, lips, and face (Figure 5) [5]. The patient was able to evaluate the expected results, express her opinion, and approve the final shape of her new smile.

There are many types of possible treatments for dental reanatomization, including composite resins and veneers with or without dental preparation. Composite resins may initially surpass the patient's expectations. However, their short-term color stability (pigmentation) and their low resistance to wear and tear (loss of shine and texture and the accumulation of bacterial biofilm) may have a negative impact on satisfaction. In a study of 180 samples of three kinds of veneers (direct or indirect resin and porcelain) cemented onto the front teeth, patients treated with porcelain restorations were significantly more satisfied after 2 years. Porcelain veneers, when made in accordance with proper indications and a precise clinical protocol, offer excellent longevity and appearance [6]. In their evaluation of 318 porcelain veneers cemented in 84 patients, [6] observed a 93.5% survival rate after 10 years. The main cause of failure was fracture of the porcelain. Bruxism and nonvital teeth significantly reduced the clinical lifespan. In a systematic literature review, the main complications found after 5 years were marginal pigmentation and loss of margin integrity [7].

As the name itself suggests, the main difference between veneers with and without preparation is the wear on the healthy dental structure [3]. The literature describes different types of preparation for porcelain veneers [8]. The techniques basically vary in terms of the level of the wear and how the incisal edge evolves. Although there is no evidence as to which preparation technique produces better clinical results, we know that veneers cemented onto dental preparations restricted to the enamel have a longer lifespan [9]. Currently, minimally invasive dentistry encourages avoiding wear on healthy tissue as much as possible. Treatments with porcelain veneers should create only the space needed to provide resistance for the restorative material (0.2–0.3 mm) [10]. In teeth where the color needs to be changed, thicker restorations may be necessary to cover the dental substrate. Thus, the diagnostic wax-up (Figure 5) and the mock-up (Figure 7) are valuable tools to determine the need for and depth of the dental preparation [11]. They represent what we hope to achieve and should be the main focus of all of the planning.

Given the health of dental elements and the patient's preoccupation with wear and tear, we proposed manufacturing porcelain veneers without dental preparation and using ceramic fragments to modify their shape. This type of treatment is indicated in situations in which the dental structure allows for material to be added, including enlargement of the incisal edge or vestibular volume, diastema closure, abfractions, gum recessions, and occlusal restorations to increase the vertical dimensions, since they do not modify or create an overcontour [2, 12]. The main contraindication for this approach is if there is no way to achieve the desired shape just by the addition of restorative material without tooth preparation. This approach is also contraindicated if the dental substrate has darkened; the minimal thickness of no-prep veneers cannot mask color alterations of more than two tones above the scale [13]. Depending on the shape of the natural teeth, a minimal dental preparation may be necessary to eliminate retentive areas and create a horizontal insertion axis for the veneer [11].

The main advantage of using veneers without preparation is the absence of wear on the teeth and, consequently, of the need to make temporary restorations. Moreover, the impression technique is simplified because there is no finish line preparation to mold. Gingival displacement is only needed when changing the emergence profile of the dental element, so that the restoration can emerge softly from the gingival sulcus, as in cases of conoid teeth or diastema closures. The main disadvantage of using veneers without preparation is the possibility of creating restorations with an overcontour and, consequently, causing gum inflammation.

The perfect combination of restorative material and cementation strategy will determine the clinical success of a restoration [14]. An adhesive cementation technique is fundamental to retain the veneers, given that they lack preparation for mechanical retention. Thus, silica-based ceramics (feldspathic porcelains, leucite-reinforced ceramics, and lithium disilicate ceramics) are indicated when making veneers. These ceramics are acid sensitive, present high translucency, and can be used in very small thicknesses [2]. In addition to favoring retention, the precementation chemical treatment by acid etching and silanization reduces the internal propagation of cracks, increasing the resistance of the ceramic to postcementation fracture [9]. The precementation chemical treatment described in this case is indicated for feldspathic porcelains. This type of ceramic has a specific working protocol, with small differences in the time required for acid conditioning [14].

## 4. Conclusion

No-prep veneers and ceramic fragments are an excellent rehabilitative option for situations in which the dental elements are healthy and can be modified exclusively by adding material and the patient does not want to suffer any wear on the teeth. Treatment success depends on the perfect interaction between the patient, clinician, and dental technician. The patient's wishes need to be transmitted by the clinic to the technician, who will make them concrete through a diagnostic wax-up. Before any restoration procedure begins, the patient should be asked to evaluate and approve the wax-up based on the mock-up made by the clinician.

## Figures and Tables

**Table 1:** Treatment of the ceramic surface before cementation.

Step	Procedure
1	Etching with 10% hydrofluoric acid for 90 seconds
2	Thorough washing with water for 1 minute
3	Drying
4	Application of the silane agent
5	Waiting 1 minute for the silane to evaporate
6	Application of the adhesive (no light activation)

**Table 2:** Treatment of the dental surface and cementation.

Step	Procedure
1	Cleaning of the dental surface with pumice paste and water
2	Protection of the adjacent teeth with thread-seal tape
3	Etching with 37% phosphoric acid for 30 seconds
4	Washing with water and air jet for 1 minute
5	Removal of excess water, maintaining the surface humidity
6	Application of the adhesive (no light activation)
7	Positioning of the porcelain restoration with adhesive cement
8	Light activation for 10 seconds
9	Removal of excess cement
10	Light activation for 40 seconds (vestibular and palatal surfaces)
